# Recognition and Management of Hyperkalemia-Induced Tachyarrhythmia in Pediatric Spontaneous Tumor Lysis Syndrome: A Simulation Case

**DOI:** 10.7759/cureus.107494

**Published:** 2026-04-21

**Authors:** Yasmin H Soliman, Manpreet Kochhar, Gianna Petrone, Robyn Wing, Sakina H Sojar

**Affiliations:** 1 Pediatric Emergency Medicine, Warren Alpert Medical School of Brown University, Rhode Island Hospital/Hasbro Children’s Hospital, Providence, USA; 2 Pediatric Hematology and Oncology, Warren Alpert Medical School of Brown University, Rhode Island Hospital/Hasbro Children’s Hospital, Providence, USA; 3 Emergency Medicine, Warren Alpert Medical School of Brown University, Rhode Island Hospital/Hasbro Children’s Hospital, Providence, USA

**Keywords:** anuria, hyperkalemia, simulation, tumor lysis syndrome, ventricular tachycardia

## Abstract

Introduction: Prompt recognition and treatment of hyperkalemia-induced arrhythmias in children is vital, as these can be life-threatening. Tumor lysis syndrome (TLS) can present with severe hyperkalemia, which, if untreated, may lead to renal failure, arrhythmias, or multi-organ failure. Simulation offers a safe way for providers to practice managing such acute scenarios. While prior simulations address hyperleukocytosis, few focus on metabolic complications like hyperkalemia in spontaneous TLS. This high-fidelity simulation aimed to improve participants’ confidence in recognizing and managing pediatric ventricular tachycardia due to hyperkalemia from TLS.

Methods: This prospective simulation-based study was conducted by pediatric emergency, general emergency, and hematology-oncology physicians who developed the simulation for pediatric and emergency medicine providers. Participants managed a case of a school-aged child with hyperkalemia-induced arrhythmia due to spontaneous TLS, progressing to oliguria. Conducted in a simulated ED setting with a high-fidelity manikin, the scenario required clinical interpretation and intervention. The primary outcome was participant self-reported confidence in recognizing and managing hyperkalemia-induced ventricular tachycardia in the context of TLS. Secondary outcomes included objective critical action performance metrics, including time to arrhythmia recognition, time to recognition of anuria, and differential diagnoses generated during the scenario. A structured debrief, following the Promoting Excellence and Reflective Learning in Simulation (PEARLS) framework, followed each session with content expertise provided by a pediatric hematology/oncologist. Participants completed post-simulation surveys on confidence, practice impact, and feedback. Data was analyzed descriptively, with results reported as frequencies or percentages.

Results: Ninety-two participants completed the survey. Fewer than 25% had previously managed TLS or related arrhythmias. A total of 99% (N=91) found the simulation relevant, and 83% (N=76) reported increased confidence in recognizing and managing ventricular tachycardia due to hyperkalemia.

Conclusions: This descriptive study demonstrated the effectiveness of high-fidelity simulation in improving provider confidence and preparedness for managing rare, high-acuity presentations such as spontaneous TLS with hyperkalemia-induced ventricular tachycardia. Participants valued its relevance, realism, and applicability. These findings lay the groundwork for future comparative and longitudinal research, including in situ implementation with interdisciplinary teams.

## Introduction

Cardiac arrhythmias in children are often secondary to non-cardiac primary etiologies such as impending respiratory arrest [1]. Electrolyte abnormalities, such as hyperkalemia, can also be a cause of arrhythmias in the pediatric population [2,3]. Hyperkalemia (an elevated serum potassium concentration) can be caused by a variety of presenting ailments. Prompt recognition of this abnormal heart rhythm is essential for providers, as immediate management can be lifesaving. One such diagnosis that can present with hyperkalemia is called tumor lysis syndrome (TLS), caused by the rapid release of intracellular components from the lysing of malignant cells. ​​With prompt recognition and appropriate management, TLS can have an overall good prognosis [4]. However, untreated, TLS can lead to severe complications and even death, due to renal failure, cardiac arrhythmias, or multi-organ failure. TLS is a well-recognized oncologic emergency in pediatric and adult populations, and most commonly occurs after the initiation of therapy for malignancies that are very responsive to treatment. However, spontaneous TLS is rare and typically occurs at diagnosis in malignancies with high apoptotic rates, such as leukemia or Burkitt lymphoma, leading to underrecognition or delayed diagnosis.

Simulation is an educational tool that allows learners to develop the skills and knowledge necessary for managing acutely ill patients in a safe learning environment. Several simulation-based educational cases have addressed pediatric oncologic emergencies, including febrile neutropenia and hyperleukocytosis [5-7]. However, to our knowledge, no published simulation cases specifically address the metabolic complications of spontaneous TLS, and more specifically, their contribution to cardiac arrhythmias and renal failure as in this acute clinical scenario.

Here, we offer a simulation case (Appendix A) of a school-aged child who presents with several days of fever and fatigue, with a more recent onset of palpitations. The patient is immediately found to be in an abnormal heart rhythm due to hyperkalemia and eventually noted to be oliguric and in likely need of dialysis. The case was targeted toward providers in emergency medicine (EM) and pediatrics, and aimed to elicit an understanding of clinical manifestations of TLS, as well as focus on management strategies.

The primary educational objective of this simulation was to improve participant confidence in recognizing and managing hyperkalemia-induced ventricular tachycardia in the setting of pediatric spontaneous TLS. Secondary objectives included enhancing provider knowledge of TLS metabolic complications and their clinical consequences, reinforcing the indications for aggressive fluid resuscitation and dialysis in the context of acute renal failure, and evaluating the educational effectiveness of high-fidelity simulation-based training for this clinical scenario.

While the learning objectives emphasize clinical recognition and management, self-reported confidence was selected as the primary outcome measure given the descriptive nature of this study and its focus on characterizing providers' perceived preparedness for this rare clinical scenario. By having the opportunity to witness this case presentation in a standardized/high-fidelity patient, we hoped that providers could confidently recognize and manage this rare, highly acute presentation in real-life clinical practice.

## Materials and methods

Development

This simulation case was created collaboratively by physicians in Pediatric Emergency Medicine (PEM), EM, and Pediatric Hematology/Oncology as part of scheduled PEM and EM didactic sessions. The case’s critical actions were designed to help facilitators identify gaps in participants’ skills and knowledge, documented during the simulation and reviewed in the debrief. Participants were expected to have prior knowledge in recognizing and managing abnormal vital signs, physical examination findings, and respiratory distress. They were also required to interpret laboratory and imaging results to diagnose monomorphic ventricular tachycardia and hyperkalemia.

Equipment/environment

The simulation took place in a mock emergency department resuscitation bay within a medical simulation center. A high-fidelity manikin (Resusci Junior, Laerdal Medical, Stavanger, Norway) dressed in a gown was used, with vital signs displayed on a monitor using Laerdal’s LLEAP software (Laerdal Medical, Stavanger, Norway). During the scenario, learners could request laboratory results and diagnostic studies, such as chest radiographs and electrocardiograms. Institution-specific resources were available, including the Broselow Tape, pediatric medication reference book, pediatric intubation cognitive aid, and intubation checklist. Pediatric simulation equipment and medication vials were available to participants.

Inclusion/exclusion criteria

The targeted population included any person who provides medical care for children in an EM setting and attends scheduled didactic sessions where the simulation will be implemented. These include attending physicians, advanced practice practitioners, fellow physicians, resident physicians, or medical students. Emergency department providers who do not attend didactics were excluded. Participation in the simulation itself was mandatory as part of their didactic session, but participation in the study was voluntary. It was explained to all participants that they could elect not to complete a post-simulation survey (Appendix B) if they did not wish to be part of the study. Those who participated in the simulation but did not complete a post-simulation survey were excluded. 

Personnel

A minimum of four personnel were required for the simulation: the facilitator, the simulation technician, a confederate facilitator portraying the nurse, and another portraying the parent. Each case began with one direct participant and several observers, who transitioned into active roles as directed by the team leader and evolving case needs. All facilitators were attending or fellow physicians in PEM, EM, or pediatric hematology/oncology.

Before the session, the faculty instructor and confederate facilitators reviewed the simulation case and debriefing guide (Appendix C). They prepared to address key topics, including the medical management of TLS and its associated complications, including electrolyte abnormalities, renal failure, and cardiac effects (Appendix D). All personnel were expected to use the case’s learning objectives and critical actions to guide progression, adapt teaching to the allotted time, and scaffold learning by prompting based on each participant’s level of clinical experience, as outlined in the case and debrief. Confederate facilitators were oriented to the case and trained on scripted prompts and cue delivery to ensure consistency in how the nurse and parent roles were portrayed across groups.

Implementation

**Figure 1:**
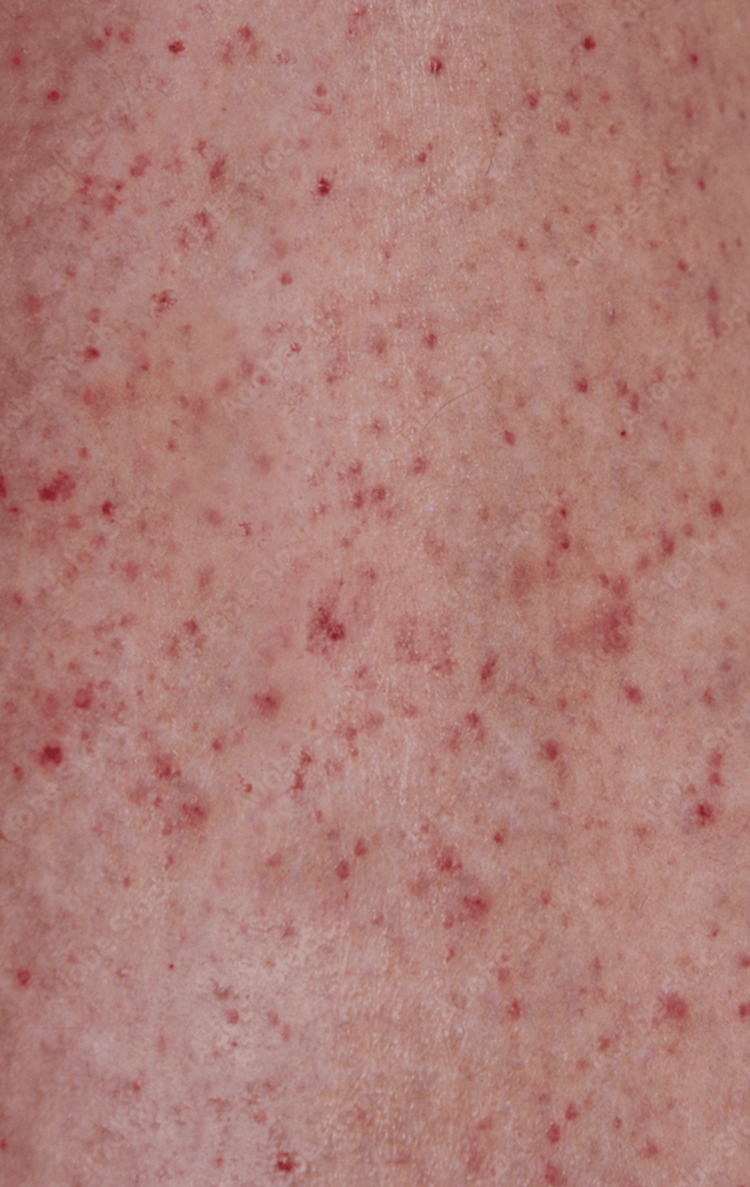
Petechial Rash Source: This is an author-owned image from the Coro Simulation Center, Brown University Health.

**Figure 2:**
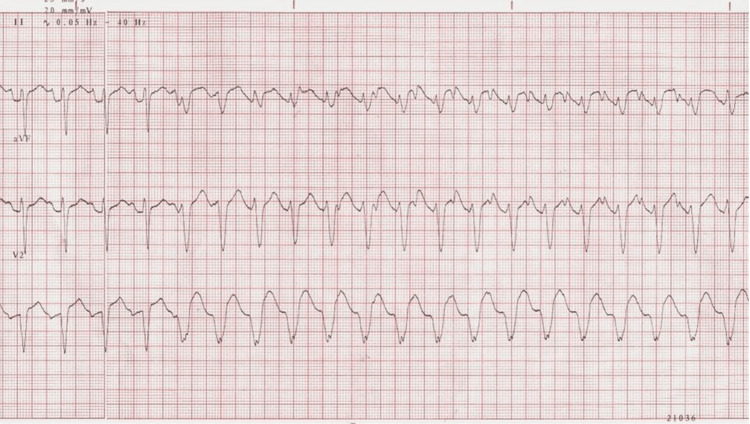
Electrocardiogram of Ventricular Tachycardia Source: CardioNetworks: Giniger. Available at: https://commons.wikimedia.org/wiki/File:Vtach_%28CardioNetworks_ECGpedia%29.jpg. Accessed May 22, 2024. Licensed under CC BY-SA 3.0 (https://creativecommons.org/licenses/by-sa/3.0/).

**Figure 3:**
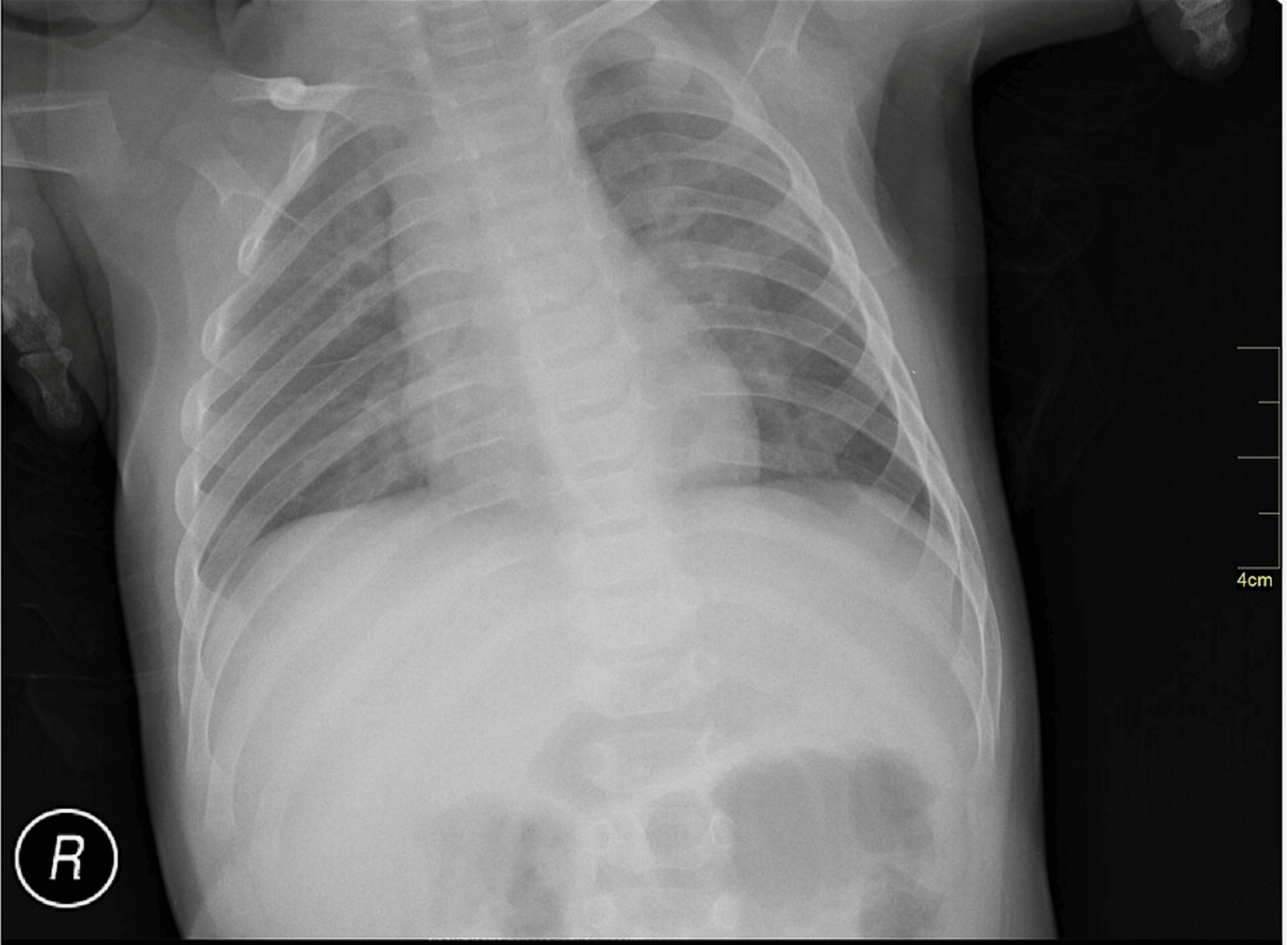
Normal Chest Radiograph Source: Nevit Dilmen. Available at: https://commons.wikimedia.org/wiki/File:Medical_X-Ray_imaging_MLK05_nevit.jpg. Accessed May 22, 2024. Licensed under CC BY-SA 3.0 (https://creativecommons.org/licenses/by-sa/3.0/).

The simulation was conducted during scheduled pediatric and EM resident didactics, with 11 groups participating. Each case lasted 15 minutes, followed by a 15 to 20-minute debrief. All learners in each group had the opportunity to lead the simulation, and volunteers to lead were requested at the start of each session. Team leaders were advised to call upon seated observers to participate in the case as needed for additional assistance. Each simulation group consisted of participants across various levels of training, including trainees at different stages, who were encouraged to function collaboratively as a team throughout the scenario. At the outset, learners were told the patient was a 10-year-old girl brought in by her parents after several days of fever, with increasing weakness and fatigue. The manikin displayed vital sign abnormalities when connected to the monitor and had a petechial rash (Figure 1), prompting learners to obtain laboratory values (Table 1) and imaging studies, such as blood gas, electrolytes, CBC, electrocardiogram (Figure 2), and chest radiograph (Figure 3). The patient initially presented with ventricular tachycardia, which appeared on the monitor and on an ECG.

**Table 1:** Laboratory Values pH: potential of hydrogen; pCO_2_: partial pressure of carbon dioxide; pO_2_: partial pressure of oxygen; BE: base excess; HCO_3_⁻: bicarbonate; Na^+^: sodium; K^+^: potassium; iCa: ionized calcium; WBC: white blood cell count; Hgb: hemoglobin; Hct: hematocrit; BUN: blood urea nitrogen; Cl⁻: chloride; Cr: creatinine; ALT: alanine aminotransferase; AST: aspartate aminotransferase; PT: prothrombin time; aPTT: activated partial thromboplastin time; INR: international normalized ratio; Mag: magnesium; PO_4_: phosphate; LDH: lactate dehydrogenase

Lab Test	Value	Pediatric Reference Range
iSTAT venous gas pH	7.31	7.35-7.45
iSTAT venous pCO_2_	30 mmHg	35-45 mmHg
iSTAT venous pO_2_	90 mmHg	80-100 mmHg
iSTAT venous BE	-7 mmol/L	-2 to +2 mmol/L
iSTAT venous HCO_3_-	18 mmol/L	22-26 mmol/L
iSTAT venous lactate	5 mmol/L	0.5-2.2 mmol/L
iSTAT venous Na+	132 mmol/L	135-145 mmol/L
iSTAT venous K+	9 mmol/L	3.5-5.0 mmol/L
iSTAT venous iCa	0.9 mmol/L	1.12-1.32 mmol/L
iSTAT venous glucose	150 mg/dL	70-100 mg/dL
WBC	81,000/µL	5,000-10,000/µL
Hgb	8.2 g/dL	11-16 g/dL
Hct	25%	33-45%
Platelets	30,000/µL	150,000-400,000/µL
Na+	132 mEq/L	135-145 mEq/L
K+	9 mEq/L	3.5-5.0 mEq/L
BUN	60 mg/dL	5-18 mg/dL
HCO_3_-	18 mmol/L	22-26 mmol/L
Cl-	98 mmol/L	98-106 mmol/L
Cr	2.8 mg/dL	0.3-0.7 mg/dL
Glucose	150 mg/dL	70-100 mg/dL
ALT	120 IU/L	7-40 IU/L
AST	100 IU/L	15-50 IU/L
Albumin	3.3 g/dL	3.5-5.0 g/dL
Calcium	6.8 mg/dL	8.8-10.8 mg/dL (4.4-5.4 mEq/L)
PT	16 seconds	11-13.5 seconds
aPTT	40 seconds	30-40 seconds
INR	1.3	0.8-1.1
Mag	1.6 mg/dL	1.5-2.3 mg/dL
PO_4_	8 mg/dL	4.0-7.0 mg/dL
Uric acid	14 mg/dL	2.0-5.5 mg/dL
LDH	410 U/L	150-300 U/L

**Figure 4:**
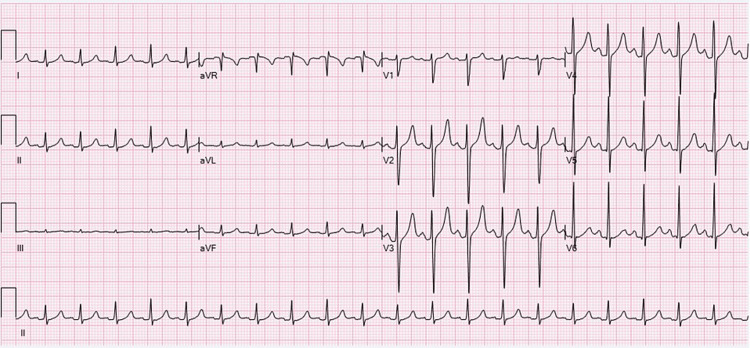
Electrocardiogram of Sinus Tachycardia Source: Ewingdo. Available at: https://commons.wikimedia.org/wiki/File:ECG_Sinus_Tachycardia_125_bpm.jpg. Accessed May 22, 2024. Licensed under CC BY-SA 4.0 (https://creativecommons.org/licenses/by-sa/4.0/).

Learners were expected to identify the abnormal rhythm, treat the hyperkalemia, and recognize renal failure in the context of TLS. The scenario was designed to highlight pediatric ventricular tachycardia secondary to hyperkalemia from spontaneous TLS. If the participants addressed the hyperkalemia with calcium gluconate and one additional appropriate intervention, the ventricular tachycardia resolved. Participants were then expected to discuss their differential diagnosis for hyperkalemia while awaiting CBC results. If participants requested a repeat ECG after treating hyperkalemia with a subsequent resolution of the ventricular tachycardia, they were presented with an ECG of sinus tachycardia (Figure 4). They were then expected to recognize the leukocytosis and renal failure, clueing them to a diagnosis of TLS and the need for dialysis. The case ended after participants admitted the patient to the pediatric intensive care unit or at the 15-minute mark. If participants did not recognize the abnormal rhythm or begin to manage hyperkalemia, the nurse would prompt participants by asking questions tailored towards achieving the learning objectives.

Debriefing

Immediately after the simulation, facilitators conducted a structured debrief using a standardized debriefing guide developed by the study team (Appendix C). The guide specified key discussion prompts, target learning objectives for each debriefing phase, and suggested language aligned with the debriefing-with-good-judgment approach to promote consistency across all sessions. The session began with a brief introduction outlining the ground rules, fostering a respectful and safe environment for effective learning and participation. The remainder of the debrief followed the multiphase format of the Promoting Excellence and Reflective Learning in Simulation (PEARLS) debriefing tool [8,9], including the reactions, analysis, and summary phases, using the debriefing-with-good-judgment approach [10-12]. During the analysis or medical management phase, a pediatric hematology/oncology specialist participated in the debrief to provide essential learning points on pediatric tumor lysis management and answer participant questions. Lastly, the summary phase encouraged learners to highlight their take-home points and gave facilitators a final opportunity to ensure that learning objectives had been covered.

Assessment

The primary outcome of this study, assessed by the post-simulation survey, was participant self-reported confidence in recognizing and managing hyperkalemia-induced ventricular tachycardia in the context of TLS. Secondary outcomes included objective critical action performance metrics documented by an observer throughout the case, including differential diagnoses discussed by the team during the case, time to arrhythmia recognition, and time to inquiry about urine output or recognition of anuria. At the conclusion of the session, learners scanned a QR code to access and complete the web-based, post-simulation survey, developed by the authors using the Qualtrics survey tool (Qualtrics LLC, Provo, UT, USA). The survey was developed through an iterative process by the multidisciplinary author team, with survey items reviewed for content validity by both the simulation educators and subject matter experts in PEM and hematology-oncology. The instrument was piloted to a group of PEM fellows who were not included in the study prior to implementation and revised for clarity.

The survey assessed participants’ perceptions of the simulation’s effectiveness in meeting its educational objectives. It included 5-point Likert-scale items evaluating whether the objectives had been achieved, as well as open-ended questions inviting participants to reflect on how the case might impact their clinical practice and to suggest improvements to the simulation or overall learning experience.

## Results

A total of 92 participants were enrolled in the study and completed the post-simulation survey. A total of 95% (n=87) of participants identified as physicians, and 58% (n=53) of participants work in EM as their primary specialty (Table 2). Five non-resident participants completed the survey, including two medical students, two EM physician assistants, and one family medicine nurse practitioner. Participant feedback regarding the quality of the case and relevance to their work is listed in Table 3.

**Table 2:** Participant Demographics PGY: postgraduate year

Participant Level of Training and Specialty	Number of Participants (N=92), N (%)
PGY-1 Resident (Total)	28 (30.4)
Emergency Medicine	14 (15.2)
Pediatrics	14 (15.2)
PGY-2 Resident (Total)	22 (23.9)
Emergency Medicine	11 (11.9)
Pediatrics	11 (11.9)
PGY-3 Resident (Total)	26 (28.2)
Emergency Medicine	15 (16.3)
Pediatrics	11 (11.9)
PGY-4 Resident (Total)	11 (11.9)
Emergency Medicine	10 (10.9)
Medicine/Pediatrics	1 (1.1)
Medical student	2 (2.2)
Physician Assistant - Emergency Medicine	2 (2.2)
Nurse Practitioner - Family Medicine	1 (1.1)

**Table 3:** Participant Feedback on Case Quality and Relevance to Their Work (N=92) * Rated on 5-point Likert scale (1=Strongly disagree, 3=Neither agree nor disagree, 5=Strongly agree)

Please Rate Your Agreement With the Following Statements:		Median*	Respondents Who Answered “Agree” or “Strongly Agree,” N (%)
This simulation case provided is relevant to my work.	Strongly Agree (5)		91 (99)
This simulation case was realistic.	Agree (4)		88 (96)
The debrief created a safe environment.	Strongly Agree (5)		91 (99)
The debrief promoted reflection and team discussion.	Strongly Agree (5)		91 (99)

A total of 22% (N=20) of respondents reported having encountered spontaneous tumor lysis in a real-life patient scenario, and 24% (N=22) encountered monomorphic ventricular tachycardia due to hyperkalemia in a real-life patient scenario before this simulation case (Table 4). Seventy-nine percent (N=73) of participants reported that they did not know the process for initiating renal dialysis at the children’s hospital (Table 4). The average time to inquire about the patient’s urine output for participants who performed this critical action was 11 minutes and 20 seconds into the simulation case. After participating in the session, 75% (N=69) of participants felt confident or very confident in their ability to recognize the need for possible dialysis to treat hyperkalemia in an anuric patient (Table 5). Participants provided written feedback on learning points they took away from the session, with examples shown in Table 6.

**Table 4:** Assessment of Participants’ Knowledge or Experience Before the Simulation Case (N=92)

Question	Respondents Who Answered "Yes," N (%)
Have you encountered spontaneous tumor lysis in a real-life patient scenario?	20 (22)
Have you encountered monomorphic ventricular tachycardia due to hyperkalemia in a real-life patient scenario?	22 (24)
Were you specifically concerned about anuria secondary to renal injury during this case?	55 (60)
Did you know the process to initiate renal dialysis at the children’s hospital?	19 (21)

**Table 5:** Participant Self-Reported Confidence Levels After Simulation Case (N=92) * Rated on 5-point Likert scale (1=Strongly disagree, 3=Neither agree nor disagree, 5=Strongly agree)

Question: After Participating in This Session, How Confident Are You in Your Ability to:	Median*	Respondents Who Answered “Confident” or “Very Confident,” N (%)
Recognize monomorphic ventricular tachycardia on a monitor in a patient with hyperkalemia and follow the appropriate protocols to manage this.	4	76 (83)
Manage hyperkalemia in a patient with new-onset leukemia.	4	69 (75)
Recognize the need for possible dialysis to treat hyperkalemia in an anuric patient.	4	69 (75)
Demonstrate efficient and effective communication skills and resource utilization in the emergency department setting.	4	71 (78)

**Table 6:** Learning Points Reported by Participants in the Post-simulation Survey Form

Inquiry About Urine Output	Diagnosis of Pediatric Tumor Lysis Syndrome	Treatment of Tumor Lysis Syndrome
"Importance of monitoring urine output in children"	“To think broadly when seeing a pediatric patient with arrhythmia”	"The importance of fast fluid for tumor lysis syndrome"
"Always ask about urine in children"	“Spontaneous tumor lysis is possible”	"Ask about urine output when considering TLS, and consult renal early for dialysis"
"Thinking about urination early on"	-	"The extent of renal injury secondary to acute TLS"

## Discussion

The case was designed for EM and pediatric providers to reinforce recognition of the clinical manifestations of ventricular tachycardia and TLS, as well as to emphasize appropriate management strategies. The simulation aimed to improve providers’ comfort and knowledge in managing hyperkalemia-induced ventricular tachycardia in the setting of spontaneous TLS. By participating in a high-fidelity simulation of this critically ill and medically complex presentation, participants reported increased confidence in recognizing and managing similar challenging cases in real-world clinical practice.

Children presenting for emergency care are more likely to present to non-children’s hospitals, with only ~20% of pediatric ED visits occurring at a pediatric hospital [13]. Exposure of critically ill children among general EM providers is variable, and studies suggest these providers often feel less prepared for pediatric resuscitation [14]. Furthermore, significant differences in the quality of pediatric resuscitative care exist across EDs [15]. Both the American Academy of Pediatrics and the American College of Emergency Physicians emphasize that improving pediatric readiness in all EDs is paramount to optimizing outcomes for critically ill children [16,17]. Simulation serves as a vital tool to help providers recognize and manage high acuity, low frequency diagnoses.

EM providers are more accustomed to evaluating ventricular tachycardia in adults, where ischemic or structural heart disease and other chronic comorbidities are common etiologies [18]. Pediatric cases of ventricular tachycardia, however, are rare and often arise from idiopathic causes, congenital heart defects, myocarditis, or, as highlighted in this simulation, metabolic derangements [19]. By exposing EM providers to a pediatric-specific differential diagnosis and management pathway for ventricular tachycardia, this simulation broadened their diagnostic framework and enhanced readiness for pediatric resuscitations that fall outside their routine scope of practice, thus improving patient outcomes. Additionally, rather than tailoring objectives to individual training levels, the learning objectives were intentionally held constant across all participants, as the simulation was designed to reflect the interdisciplinary, team-based nature of real clinical encounters. We believe this approach strengthens the educational value of the simulation by mirroring actual clinical environments where providers of varying experience must communicate and manage high-acuity presentations together.

Although TLS is a well-recognized oncologic emergency in pediatric and adult populations [20-23], its spontaneous form, particularly when presenting with life-threatening arrhythmias and acute kidney injury, remains a rare and challenging diagnosis [24]. Most participants in our study had never encountered spontaneous TLS or hyperkalemia-induced ventricular tachycardia in practice, underscoring the value of targeted experiential learning to prepare clinicians to manage these high-acuity, low-frequency events. Notably, while malignancy was often included in early differentials, spontaneous tumor lysis was seldom discussed until hyperkalemia was identified on labs or after consultation with hematology.

This case was intentionally designed with multiple layers of complexity, which posed challenges for some learners. Nursing prompts were occasionally necessary to advance the case and ensure learning objectives were met, for example, encouraging teams to order labs, consider differential diagnoses for hyperkalemia, or engage consultants. These prompts mirrored real-world practice, where nursing team members often provide timely input during resuscitations (e.g., “Doctor, would you like me to obtain a blood gas?” or “Why is the potassium so elevated?”). This approach maintained realism while supporting learner development. This complexity was also a strength of the simulation as it addressed multiple educational opportunities for learners, including hyperkalemia, acute renal failure from other etiologies, as well as reinforcing the Pediatric Advanced Life Support Algorithms. The simulation also reflected the importance of multidisciplinary involvement, as highlighted by pediatric readiness guidelines. The simulation and survey included advanced practice providers, such as physician assistants, nurse practitioners, and medical students, highlighting the strength of team-based training.

Although the definitive diagnosis of TLS was not immediately apparent during the case, participants were required to recognize critical illness, initiate treatment for hyperkalemia, and escalate care based on clinical deterioration. This diagnostic ambiguity is a strength of the case design, encouraging learners to make decisions based on physiologic derangement and patient stability rather than diagnostic clarity, a common scenario in real-world clinical practice. Many participants cited this uncertainty as a key strength of the simulation during the debriefing.

Acute renal failure from hyperuricemia is a known complication of TLS [25], particularly in patients with large tumor burdens, such as acute lymphoblastic leukemia or lymphomas [26-28]. Nearly 80% of participants reported not knowing the dialysis initiation process at their institution before the simulation, an actionable knowledge gap with direct implications for patient care. Learners also highlighted critical takeaways, including the importance of assessing urine output early in the diagnostic process, selecting appropriate fluids in TLS, and involving nephrology consultants early. This was reflected by the objective data showing the average time to inquire about urine output was 11 minutes and 20 seconds in only two groups that performed the critical action. Additionally, some participants did not recognize the clinical manifestations of this patient’s acute renal failure with anuria and ordered potassium-containing fluids (lactated Ringer's) before receiving laboratory results that revealed hyperkalemia and elevated creatinine. This was discussed during the debrief, underscoring the importance of assessing both renal function and hydration status through urine output history, an easily obtainable clinical history detail, especially in patients requiring fluid resuscitation, rather than waiting for lab confirmation. Even after noting hyperkalemia on lab results, no group immediately asked about urine output to assess renal function. This omission provided a valuable teaching moment on how early clinical assessment of pediatric urine output can guide safer, more effective management of the critically ill patient.

Limitations

Despite its several strengths, this study has some limitations to note. It was conducted at a single academic medical institution and at a children’s hospital, which may limit the generalizability of findings to other institutions that have different pediatric readiness, resources, protocols, and/or learner populations. Results were obtained through survey data of self-reported confidence, which is inherently subjective and may be influenced by recall bias, social desirability bias, or the tendency for participants to overestimate confidence and abilities immediately after an educational intervention. Though there was a recording of time to critical action (inquiry of urine output), the results lacked more objective clinical performance data. Conclusions are thus limited to perceived preparedness rather than competency. In an effort to prevent priming learners on the case content prior to participation, a pre-simulation survey was not distributed, making pre- and post-knowledge assessment difficult.

Future implementations may include retrospective pre- and post-confidence assessments, objective clinical performance metrics (such as time to recognition of hyperkalemia or fluid choice), and longitudinal integration to better assess learner competency and retention. Expanding the simulation to in situ settings with broader participation from multidisciplinary team members, including emergency department nursing staff and social workers, would accurately reflect the dynamics of real-time pediatric resuscitations and further enhance team-based communication and interprofessional collaboration. Expanding the simulation across community emergency departments and general hospitals would allow for broader validation of its effectiveness and facilitate comparisons across different training environments, to help establish standardized educational approaches for rare but high-risk pediatric emergencies, like spontaneous TLS. Further studies could explore whether this simulation translates to improved clinical outcomes for pediatric patients, particularly in the early recognition and management of oliguria or anuria.

## Conclusions

This study highlights the value of high-fidelity simulation as an essential tool in medical education, particularly for low-frequency, high-acuity presentations such as spontaneous TLS with monomorphic tachycardia due to hyperkalemia. While only a minority of participants had prior experience managing this diagnosis, most reported increased confidence in recognizing and treating TLS and ventricular tachycardia, as well as in their communication skills and team dynamics following the simulation. Nearly all participants found the simulation highly relevant to their clinical practice, realistic, and conducted in a psychologically safe environment. High-fidelity simulation provides a critical platform for practicing diagnostic reasoning and life-saving interventions in rare, high-risk scenarios that often present subtly yet progress rapidly. By enhancing provider preparedness for complex clinical cases, simulation promotes timely diagnosis, improves patient safety and outcomes, and strengthens team-based resuscitation efforts.

Future directions include expanding the simulation to a more interdisciplinary format by incorporating nurses, respiratory therapy, social work, and other ancillary staff members involved in resuscitation and who are stakeholders in pediatric readiness. Additionally, creating a longitudinal curriculum may help reinforce learning objectives, assess for knowledge and skills retention, and sustain the increased confidence as reported by participants.

## Figures and Tables

**Figure 5:**
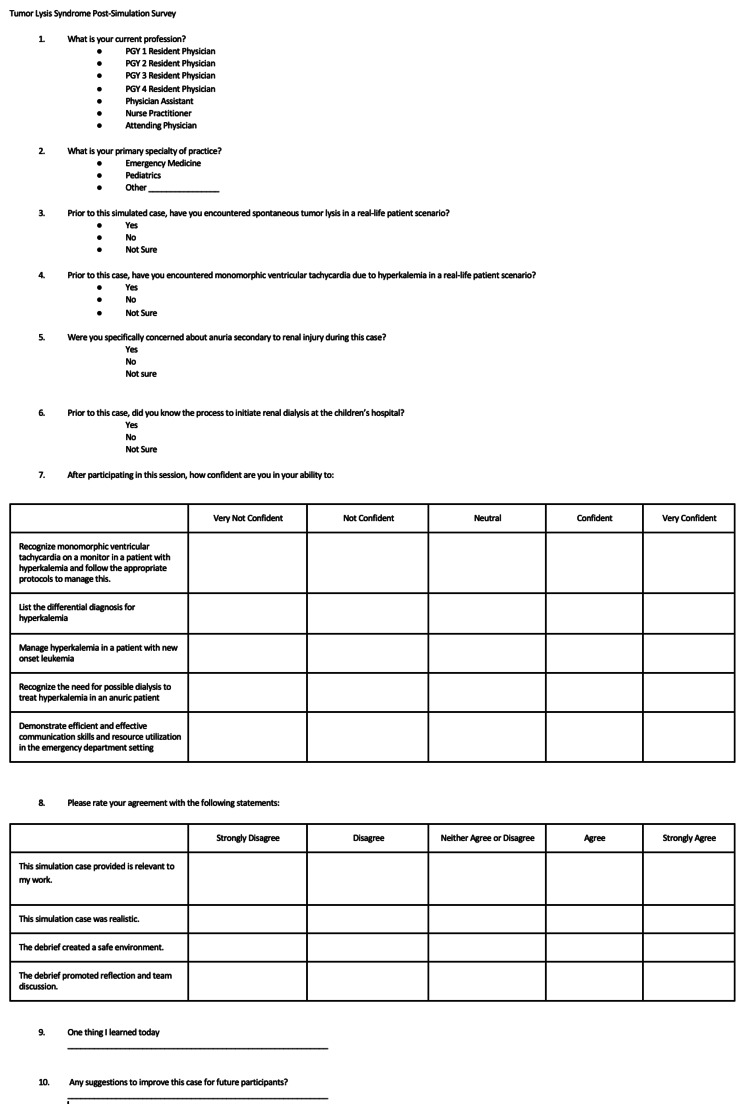
Post-simulation Survey

## Appendices

Appendix A 

**Table 7:** Tumor Lysis Simulation Case and Branch Points

SIMULATION CASE TITLE: The K Is Not Okay: Hyperkalemia in Spontaneous Pediatric Tumor Lysis Syndrome	
PATIENT NAME: Kyleigh PATIENT AGE: 10 years old CHIEF COMPLAINT: Weakness, lethargy, fevers, and palpitations PHYSICAL SETTING: Critical Care Bay in Emergency Department	
Brief Narrative Description of Case	10 yo F brought to the trauma room with weakness, lethargy, and palpitation in the setting of having a fever over the last 10 days. She had been diagnosed with a viral illness the week prior. The monitor shows monomorphic ventricular tachycardia, which learners should determine is secondary to hyperkalemia. Learners should work through the stabilization of a patient with hyperkalemia, consider the differential diagnosis of hyperkalemia, recognize that the patient has signs of new-onset leukemia, and recognize that hyperkalemia is likely secondary to tumor lysis syndrome. More advanced learners may consider initiating dialysis therapy after noting the patient is anuric due to acute kidney injury.
Primary Learning Objectives	Recognize monomorphic ventricular tachycardia on a monitor in a patient with hyperkalemia and follow the appropriate protocols to manage this. List the differential diagnosis for hyperkalemia, including tumor lysis syndrome, in a patient with new-onset leukemia. Manage hyperkalemia in a patient with new-onset leukemia. Recognize the need for dialysis to treat hyperkalemia in an anuric patient. Demonstrate efficient and effective communication skills and resource utilization in the emergency department setting
Critical Actions	1. Place the patient on the monitor, and recognize an irregular rhythm. 2. Verbalize abnormal vital signs, presence of monomorphic ventricular tachycardia with a pulse. 3. Perform a physical exam and note the presence of pallor, petechiae, and jaundice. 4. Ask for IV access and initial labs, including CBC, venous blood gas, and chemistries. 5. Provide supplemental oxygen. 6. Obtain history from the patient’s family and the patient. 7. Verbalize hyperkalemia as a cause for monomorphic ventricular tachycardia. 8. Ask for placement of Zoll pads, calcium gluconate, and a 12-lead EKG. 9. Ask for consultation with pediatric hematology once CBC reveals leukemia. 10. Recognize abnormal creatinine, anuria, and verbalize IV fluids to be administered at 1.5-2x maintenance rate with possible need for dialysis 11. Obtain tumor lysis labs once CBC reveals leukemia 12. Admit to pediatric ICU
Learner Preparation or Prework	Kyleigh is a 10-year-old female who was brought in from home by her parents. They note that she has had a fever for 10 days and has become increasingly weak, tired, and ill-appearing. Today, she was complaining of palpitations as well. She appears pale and diaphoretic.

Initial Presentation			
Initial Vital Signs	HR: 160 | BP: 112/74 | RR: 24 | T: 39 C | Oxygen Sat: 96% on RA		
Overall Setting and Appearance	The patient appears ill, weak, and diaphoretic. She is pale. She is speaking and is extremely uncomfortable. She says that her heart is racing. No monitors are on the patient. Exposure of the mannequin reveals diffuse petechiae to the chest, abdomen, back, upper extremities, and lower extremities.		
Standardized Participants (and Their Roles in the Room at Case Start)	Bedside Nurse: This nurse is appropriately concerned, helpful, and knowledgeable. Only those interventions requested by the learner(s) should be performed. If learners do not ask about placing the patient on the monitor or note the abnormal rhythm, the nurse may need to point it out. The nurse may also need to note the petechial rash if not noted by the learners in a timely fashion, pointing towards the diagnosis of malignancy. Medication Nurse: This nurse will prepare medications, call for extra personnel, and relay laboratory and imaging results as the learner (s) request critical actions. Parent/Caregiver: This person can provide a history of present illness, past medical history, and events leading up to presentation in the emergency department. Faculty Instructor: The faculty instructor observes the performance of the learner(s), provides feedback and instruction to the bedside nurse to facilitate case progression, and facilitates the debriefing session. They will be present in the simulation control room to help guide the case and various branch points.		
HPI	Information volunteered by the parent: “Kyleigh has been sick for the last two weeks, it really seems like she's always sick. She’s had about 10 days of fevers. We took her to the pediatrician and they did a covid/flu/rsv test, and strep test but it was negative. They told us that she likely has a viral illness. It just seems like she’s gotten sicker and sicker. She seems like she’s going to pass out.” Information volunteered by patient: “My heart is racing” “I think I’m going to be sick” The following details will be revealed only when asked by the learner: I don’t think she’s urinated in almost two days, hard to know as she’s independent at this age		
Past Medical/Surgical History	Medications	Allergies	Family History
None	None	None	Abnormal heart rhythm in maternal grandfather.
Physical Examination			
General	Alert, but appears lethargic and weak		
HEENT	Mucous membranes dry		
Neck	Cervical lymphadenopathy present		
Lungs	Clear to auscultation		
Cardiovascular	Tachycardic, no murmur, central pulses intact, cap refill about 2-3 secs		
Abdomen	Soft, nontender, +hepatosplenomegaly		
Neurological	Answering questions appropriately, non-focal neurological exam but is feeling generally weak		
Skin	Petechiae noted to chest, abdomen, extremities		
GU	Normal/Unremarkable		
Psychiatric	Normal/Unremarkable		

Changes and Case Branch Points		
Intervention / Time Point	Change in Case	Additional Information
5 minutes into the case	BP begins decreasing slightly if no IV fluids have been given; patient remains in stable Vtach HR: 169 | BP: 100/72 | RR: 24 | T: 39 C | Oxygen Sat: 96% on RA	RN alerts the provider: “Doctor, the blood pressure is dropping”
Team requests venous iSTAT with labs	RN asks “would you like that iSTAT with electrolytes or lactate?”	RN alerts the provider: “Doctor, the K is 9 on the iSTAT”
Patient is rolled for the participant to examine the back	Participants will be notified that patient has a petechial rash if they verbally ask if patient has a rash.	Patient states: “Ouch that really hurts my leg!”
Epinephrine is given by intramuscular injection for suspected anaphylaxis	Patient heart rate increases by 20 beats per minute over next 1 minute. Respiratory rate decrease to 16 HR: 180 | BP: 120/92 | RR: 28 | T: 39 C | Oxygen Sat: 96% on RA	
Participant requests finger stick blood glucose.	Glucose level is 150	
Antibiotics are given for suspected sepsis	Parent may ask “what are you giving her? she has an infection? Is that what’s causing her fevers?” prompting learner to consider other differentials	
EKG performed for complaint of palpitations		EKG will show monomorphic ventricular tachycardia
Team implements at least 2 interventions for hyperkalemia management, must include calcium gluconate + one other intervention (albuterol, bicarb, insulin + glucose)	Ventricular tachycardia will resolve HR: 96 | BP: 116/78 | RR: 24 | T: 39 C | Oxygen Sat: 96% on RA	
Team cardioverts patient	Patient will say “Mom what are they doing, noo” Mother will ask “Is that necessary?”	No change; Ventricular tachycardia will not resolve
Team administers any antiarrythmics		No change; Ventricular tachycardia will not resolve
If no EKG obtained or rhythm not identified 10 minutes into case		Patient will continue to complain of heart racing. RN will also suggest abnormality on monitor
Learner may request a urine culture for suspected sepsis.	Parent will respond with “I do not think she has been able to pee”. If chosen to cath, RN will indicate there is no urine on catheterization, prompting learner to ask about urine output	Parent will disclose that patient has not been urinating if asked by learner.
Learner may administer albuterol during management of hyperkalemia	Patient will state “My heart feels like it’s racing more”. HR: 169| BP: 116/78 | RR: 24 | T: 39 C | Oxygen Sat: 96% on RA	
Team requests hematology/oncology consult	1 minute later, consulting team will call with additional recommendations	Recommendations will include additional labs (uric acid, phos, LDH, coags), Chest X-ray, blood culture, Cefepime for fever. Will recommend IV fluids at 1.5-2x maintenance rate and rasburicase for elevated uric acid.
Team will consult Cardiology	RN will tell provider “Cardiology was consulted”	No consultant will call during the duration of the case

Appendix B

Appendix C

**Table 8:** Hyperkalemia in Tumor Lysis Syndrome Debriefing Guide

PEARLS Framework for Debriefing:										
1. Reactions phase- opportunity for learners to express their emotional experiences upon completion of the case										
2. Description phase- opportunity for learners to summarize key events in the scenario to ensure that educators and learners are on the same page; often started by asking for one-liner from participants										
3. Analysis phase- opportunity to explore the medical decisions, technical, teamwork and communication performance of the team										
4. Summary phase- review of key take home points, led by learners or educator/facilitator; Final opportunity to ensure learning objectives covered										

Debriefing with Good Judgement: Ensure feedback is supportive to staff to promote reflection and change for a more effective impact on outcome.							
- Advocacy with inquiry							
- Assume competence and good intentions							
- Mutual Learning							
Ie: “I noticed the team…..I am concerned….Can you walk me through what was going through your mind at that point?”							

General Debriefing Goals:		
Create a safe learning environment		
PreBrief by sharing a learning contract		
Normalize gaps in performance when applicable		
Ask open ended questions (avoid yes/no questions)		
Allow space for questions		

1) Reactions Phase: If a group or team member is emotionally charged (e.g. sad, mad, or frustrated), it is a priority to address those emotions to promote a safe environment for which participants are receptive to feedback throughout the debrief. It is important to validate emotions expressed by participants and encourage other participants to provide reactions as well.							
Facilitator might say:							
· “How did that feel?”							
· “How did that go?”							
· “Initial reactions to the scenario?”							
· “How are the rest of you feeling?”							
2) Description phase: Summary of key events to ensure that educator and participants are on the same page to ensure a more meaningful debrief going forward.							
Facilitator might say:							
· “Could someone summarize the case so we are all on the same page?”							
· “From your perspective, what were the main issues you dealt with?							
3) Analysis phase: Promote reflection on performance (medical decision making, technical skills, teamwork, and communication), identify opportunities for improvement. Discussion points can arise from observer notes/comments obtained on data collection sheet during the simulation.							
Facilitator might say:							
· “Let’s talk more about the case.”							
· “What aspects did your team manage well? Why?”							
· “What could your team manage better next time? Why?”							
· “I want to spend a couple minutes talking about XXX. Can you tell me more about what was going on?”							
· I noticed you [behavior]…next time you may want to [suggested behavior]… because [provide rationale].							
5) Summary phase: Opportunity to review key learning points. Participants’ or educator can identify take home points.							
Facilitator might say:							
· “This was a scenario of a patient with new-onset malignancy with spontaneous tumor lysis who was found to have ventricular tachycardia due to hyperkalemia.”							
· “Signs and symptoms of hyperkalemia include…”							
· “Formulating a list of possible diagnoses is critical to identifying an etiology and determining a treatment plan.” - sepsis, malignancy, cardiac etiology (myocarditis in setting of fever for 10 days)							
· “Evaluation of stable Vtach secondary to hyperkalemia includes: evaluating the ABCDE’s, obtaining IV access and lab work.”							
· “Management of hyperkalemia includes: Albuterol, sodium bicarbonate, Insulin + Dextrose, Kayexalate, Dialysis"							
· “Getting to that point and thinking about dialysis, was anyone specifically thinking about renal injury when assessing urine output? And did the idea of dialysis come up?”							

Appendix D: spontaneous TLS teaching points

Overview

Tumor lysis syndrome is a pattern of specific metabolic abnormalities that occurs as a result of rapid cell turnover (“tumor lysis”). It commonly develops after the initiation of therapy for malignancies that are very responsive to treatment. Spontaneous TLS is rare, but mostly seen at the time of diagnosis for malignancies which have a rapid rate of apoptosis (i.e. leukemia, Burkitt’s, aggressive NHL).

Key Facts

1. Tumor lysis syndrome consists of hyperkalemia, hyperphosphatemia, hyperuricemia, and hypocalcemia

2. Presentation is often marked by signs of metabolic/electrolyte abnormalities (lethargy, nausea, vomiting, hypotension, muscle spasm, cramps, tetany, seizures, cardiac dysrhythmia)

3. Renal dysfunction may or may not be present at time of presentation

     a. Uric acid concentrates in the renal tubules which causes precipitation and stone formation

     b. Released phosphate binds with calcium which can precipitate in the microvasculature/renal tubules

4. Lactic acidosis secondary to poor tissue perfusion may worsen hyperuricemia/renal dysfunction. This can be seen when patients have a particularly high WBC.

Key Evaluations and Management

1. BMP, Mg, Phos, Uric Acid, LDH should be obtained in all children with a suspected malignancy.

2. History should evaluate for hydration status/renal function

3. Hydration, hydration, hydration! And renal consultation! Avoid potassium-containing fluids.

4. Dialysis may be avoided with aggressive management. Ideally, fluids should be run at 1.5-2x mIVF. Lasix may need to be given to manage volume.

5. Management should focus on lowering K, Phos, and Uric acid.

     a. Albuterol, insulin, diuretics, K binders, phos binders, recombinant urate oxidase (rasburicase), xanthine oxidase inhibitor (allopurinol)

6. Close monitoring and re-evaluation are necessary in early management; consider PICU.

7. Frequency of repeat labs depends on the severity at the time of presentation.
